# Oral and inhaled sodium cromoglicate in the management of systemic mastocytosis: a case report

**DOI:** 10.1186/1752-1947-4-193

**Published:** 2010-06-26

**Authors:** Alan M Edwards, Hans Hagberg

**Affiliations:** 1 The David Hide Asthma and Allergy Research Centre, St Mary's Hospital, Newport, Isle of Wight PO30 5TG, UK; 2 Department of Oncology, University Hospital, 75185 Uppsala, Sweden

## Abstract

**Introduction:**

Mastocytosis is a rare disease consisting of a group of disorders characterized by a pathologic increase in the number of mast cells in one or more organ system. Treatment is symptomatic. Oral sodium cromoglicate (SCG) is the only treatment licensed for the treatment of mastocytosis. In this case we report how in a mastocytosis patient being treated with H_1 _and H_2 _antihistamines, and oral sodium cromoglicate, the addition of inhaled sodium cromoglicate resulted in further improvement. This is the first report of this use of the drug in this disease.

**Case presentation:**

The subject is a Caucasian woman aged 40 years. Symptoms of mastocytosis began when she was aged 13 years, but the diagnosis was not made until after her first pregnancy aged 33 years. Symptoms improved with H_1 _and H_2 _antihistamines, and oral sodium cromoglicate, but it required the addition of inhaled sodium cromoglicate to produce further improvement, specifically in the symptoms of bone pain, fatigue and headache. Doses of oral sodium cromoglicate had to be increased if challenged with a food to which the subject was sensitive. Doses of inhaled sodium cromoglicate had to be increased during the menstrual period.

**Conclusions:**

Patients suffering from the rare disease of mastocytosis have symptoms affecting many body systems. Symptoms result from the release of inflammatory mediators from mast cells. Sodium cromoglicate, a drug that reduces the release of mediators from mast cells, is effective in controlling gastrointestinal symptoms, but less effective in those affecting other body systems. In this case report we have shown that the addition of inhaled sodium cromoglicate controls the symptoms of bone pain, fatigue and headache and also that the doses have to be increased during the menstrual period.

## Introduction

Mastocytosis (SM) consists of a group of clonal disorders characterized by a pathologic increase, usually low, in the number of mast cells in one or more organ system. The most common form of SM is indolent SM (ISM) comprising around 80% of cases. Patients with SM have symptoms related to tissue responses to the release of mast cell (MC) mediators, infiltration of tissues by MC or both. MC mediator-related symptoms are heterogeneous and can appear either spontaneously or in response to certain triggers. The consequence of the release of mediators is a range of symptoms of variable severity, from mild to life threatening and can involve the skin, the gastrointestinal tract, the skeletal system, cardiovascular system, central nervous system and even induce constitutional symptoms [1].

A curative treatment of SM is not available. Pharmacological treatment is symptomatic and in most cases this consists of a rationale combination of MC blockers, such as oral sodium cromoglicate (SCG), H_1 _and H_2 _antihistamines while other therapies such as corticosteroids, and leukotriene antagonists are less frequently employed.

SCG is a chromone, initially developed as an inhaled dry powder for the treatment of asthma [2], and subsequently as a nasal spray for allergic rhinitis, eye-drops for allergic conjunctivitis and oral capsules, sachets and oral aqueous solution for food allergy. In the USA the oral solution (Gastrocrom™) is licensed for the treatment of SM.

SCG is the disodium salt of a strong acid with molecular weight 512. It is poorly absorbed through all body surfaces apart from the bronchial mucosa where the entire drug reaching the bronchial mucosa after inhalation is absorbed. The inhaled product, is available in various forms, a capsule containing 20 mg of SCG powder, metered dose inhalers (MDIs) delivering either 1 mg/puff or 5 mg/puff and aqueous solution for nebulization. Approximately 10.4% of the inhaled dose from capsules is deposited in the lung all of which is absorbed [3].

When administered orally between 0.8% and 1% is absorbed systemically. The drug is not metabolized, most is excreted unchanged during the first 24 hours after administration, half in the feces and half in the urine [4].

The first report of the use of SCG administered orally for the treatment of SM was in 1974 [5]. There have been four controlled trials of oral SCG in SM [6-9]. The dose of SCG used varied between 400 mg/day to 800 mg/day. In some of these trials the addition of SCG resulted in the improvement of both gastrointestinal and non-gastrointestinal symptoms. In others only the gastrointestinal symptoms were improved.

Symptoms such as bone pain and headache that result from mediator release from MCs in the bone marrow and meninges will require higher blood levels of SCG than that achieved by oral SCG. We report on a case using inhaled SCG to achieve these ends.

## Case presentation

### Patient

Our patient is a Caucasian woman aged 40 years. Symptoms now recognized due to SM occurred from the age of 13 years but were not severe enough to seek advice or a diagnosis. Symptoms became more severe after the birth of her first child. These consisted of body pain, stomach pains, headache, flushing, itchy spots, severe fatigue and difficulty in concentration. The diagnosis of ISM was made a year later following a bone marrow biopsy. Initially treatment was an antihistamine only. Despite this she continued to be very fatigued, with daily bone, muscle and head pain. She also had severe gastrointestinal symptoms, diarrhea and abdominal pain. At this time her serum tryptase was 49 μg/mL. She was referred to one of us (HH). At that time her treatment was changed to desloratadine 5 mg tablets, one to two daily, lanzoprazole 15 mg tablets, when needed, and acetylsalicylic acid 500 mg tablets, up to two tablets four times daily, and SCG oral solution (Lomudal GI™), 200 mg four times daily. This change in treatment resulted in an improvement in symptoms, particularly the gastrointestinal.

However, she still had symptoms specifically of bone pain and headache, it was decided to try the effects of adding inhaled SCG to her current treatment.

In order to monitor the progress of symptom control with the introduction of inhaled SCG, our patient recorded the severity each day of 21 symptoms using a daily diary card (see Additional File 1). The severity of symptoms was recorded using a zero to four scale; 0 = no symptoms, 1 = mild, 2 = moderate, 3 = severe, 4 = very severe. Completed diary cards were returned to one of us (AME) via email on a regular basis together with any comments and advice on any dosage changes of the SCG products communicated in the same way. Our patient was also seen by one of us (HH) regularly at a specialist hospital clinic. At each visit blood was taken for measurements of serum tryptase.

Inhaled SCG was administered using gelatine capsules (Lomudal Caps.) each containing 20 mg of pure SCG powder. The initial dose was 20 mg (one capsule) inhaled four times daily using an Eclipse Inhaler.

## Results

The observations reported here were made over a period of five and a half months during which the subject kept daily diary cards recording symptom severity. For the first three days she remained on existing therapy.

**Figure 1:**
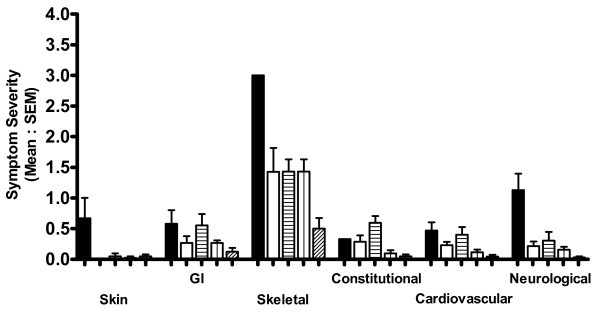
**Mean symptom scores**. Mean symptom scores recorded before the introduction of inhaled sodium cromoglicate and for the first eight weeks after introduction. Symptoms (22 in total) are grouped according to organ systems. Symptoms are recorded daily by patient using a 0-4 scale. Columns are mean scores for two weeks. Error bars are standard error of means. Key: solid black columns - before the introduction of inhaled sodium cromoglicate. First eight weeks after the introduction of inhaled sodium cromoglicate; solid white columns - weeks 1 and 2; columns with horizontal lines - weeks 3 and 4; columns with vertical lines - weeks 5 and 6; columns with diagonal lines - weeks 7 and 8.

Figure 1 shows the mean daily diary card scores for the six symptom groups both for the three days before the introduction of inhaled SCG (solid columns) and for the following eight weeks at a dose of 80 mg/day administered as 20 mg four times daily. It can be seen that there was an improvement in all symptoms, but the improvement in bone pain (Skeletal Group) was not apparent until after six weeks of treatment. Interestingly a worsening of most symptoms occurred during weeks three and four of this period, the week before and the week of her monthly menstrual period.

**Figure 2:**
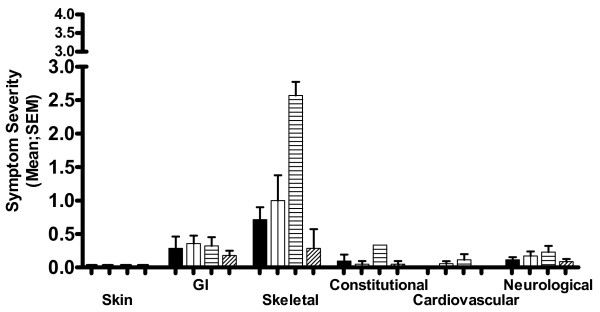
**Mean symptoms scores**. Mean symptom scores covering a four week period commencing ten weeks after starting inhaled sodium cromoglicate treatment. Dosage of inhaled sodium cromoglicate 80 mg/day throughout. Menstrual bleed occurred during Week 3 of this four-week period. N.B. 'y' axis shows 0-3 range of 0-4 scale. Key: solid black columns - Week 1; columns with vertical lines - week 2; columns with horizontal lines - week 3; columns with diagonal lines - week 4.

Figure 2 shows the mean daily symptom scores for the two weeks before her next menstrual bleed, the week of the bleed and the week following. During the week of the menstrual bleed, bone pain (Skeletal symptoms) was worse, mean ± standard deviation (SD) 2.57 ± 0.53.

On the basis of these findings she was advised to increase the dose of oral SCG on any day she ate a food which she knew might trigger a reaction; in addition to increase the dose of inhaled SCG to two capsules four times a day (160 mg/day) from just before and during the expected menstrual period.

**Figure 3:**
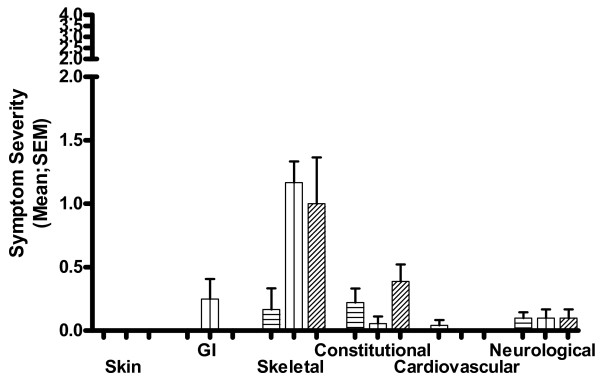
**Mean symptom scores**. Mean symptom scores covering a three-week period commencing 20 weeks after starting inhaled sodium cromoglicate treatment. Menstrual bleed occurred during week 2 of this 3-week period. Dosage of inhaled SCG: 80 mg/day during week 1, 160 mg/day during week 2, 80 mg/day during week 3. N.B. 'y' axis shows 0-2 range of 0-4 scale. Key: columns with horizontal lines - week 1; columns with vertical lines - week 2; columns with diagonal lines - week 3.

She followed this advice over her next menstrual period. As depicted in Figure 3 up to two days before her menstrual bleed she had been scoring zero for bone pain, this worsened during the days of the menstrual bleed but the increase in severity was less than when she did not increase the dose of inhaled SCG, mean (SD) score for bone pain 1.17 (0.4).

She had abdominal pain and diarrhea on one day during the menstrual bleed and increased the dose of oral SCG to 1200 mg that day; she had no pain the following day and no diarrhea after two days.

The time period covered by this report was for a five and half month period from June to November. In the spring of that year her serum tryptase was 52 μg/mL; in the autumn of that year it was 50 μg/mL and in the spring of the following year was 44 μg/mL.

Our patient has continued to use this treatment up to the time of this report.

## Discussion

In this case report we describe the management and improvement in symptoms of a patient with ISM when inhaled SCG is added to existing treatment.

Oral SCG has been used in the management of SM for a number of years, with benefit, but there has been little work examining the optimal dose. Our patient found that for most of the time a dose of 800 mg/day was adequate to control her gastrointestinal symptoms, but she had to increase the dose to 1200 mg/day should she eat something that might trigger an exacerbation. In a study in food sensitive patients it has been shown that single doses ranging from 100 to 800 mg were needed to block a reaction to food challenge [10]. Single doses of 500 mg have been used to prevent the effects of food challenge [11].

The symptoms that improved with the introduction of inhaled SCG at a dose of 20 mg four times daily were bone pain, headache and fatigue. On one occasion when our patient reduced the dose to 20 mg three times daily for two days, bone pain returned on the second day, and fatigue and headache the day after. This also happens if she forgets to increase the dose during her monthly menstrual periods.

We suggest that the worsening of specific symptoms when the dose of inhaled SCG was reduced supports that the effect observed is drug related and not a placebo effect.

The worsening of symptoms during the menstrual cycle, possibly related to MC activation has been reported in other cases of ISM. An Interactive Case Report in the *British Medical Journal *describes a woman who had hypotensive shock at the onset of menstruation as one of the symptoms of ISM [12].

We can hypothesize that bone pain results from the release of mediators from MCs in the bone marrow.

It was therefore considered necessary to try to achieve maximum blood levels of SCG. This is more likely with inhaled SCG than with increasing the dose of oral SCG. After a single oral dose of 300 mg peak blood levels of 10.75 ± 2.54 ng/mL in healthy subjects and 6.02 ± 1.48 ng/mL in food allergic patients have been reported [13].

All the published studies with inhaled SCG dry powder have been conducted using the Spinhaler. This provides peak blood levels of 42 ± 26 ng/mL in healthy volunteers [14].

In arriving at our conclusions that inhaled SCG can improve the symptoms of SM, we relied on the changes in subjective symptom severity recorded by a single patient. Whilst this is not evidence from a controlled trial we believe that patient-related outcomes (PROs) are important and useful data in deciding on the optimal use of a treatment. An editorial in *Clinical & Experimental Allergy *stated that symptom scores are an example of PROs and both the Federal Drug Administration (FDA) and the European Medicines Agency (EMEA) underwrite the importance of PROs in the study of new drugs [15]. We therefore believe that the changes reported in diary card symptom scores are reliable information confirming drug efficacy.

Serum levels of tryptase did fall over the period covered by this report. The reduction was small. It is not known if this was due to the introduction of inhaled SCG

Oral SCG alone was not adequate to control the bone pain, fatigue and headaches. It was necessary to add inhaled SCG and to double the dose during parts of the menstrual cycle for this to be achieved.

## Conclusions

SM is a rare condition that results in symptoms in multiple organ systems. Symptoms result from the release of inflammatory mediators from MCs which are present in large numbers. The release of mediators can be reduced, also reducing the severity of the symptoms by the use of SCG. In this case report we have shown that the effect of SCG is dose dependent and also dependent on the method of administration. For optimal symptom control both oral and inhaled SCG is required. This is important information for physicians dealing with this disease.

### Patient's perspective

Since I've been around 13 years old I had problems with stomach and back pains. Off and on I tried to go to the doctor to get help, but never got any other help than temporary medications. Soon I realized that going to the doctor was a waste of time since they couldn't help me either way, so I bought my medications myself on the local pharmacy and went on that way. Looking at things now I clearly can see that my illness has been worse in periods of time. Even during my teens I had longer periods with fatigue and such, which went away eventually. All this time I've been able to go to school/work though. In 2002 I got pregnant and gave birth to a child. The pregnancy was wonderful. I lost almost all my symptoms (which I then didn't know was symptoms of this) and felt very healthy and energized, with no pain stomach problems. After giving birth I felt well during the first months but after that things went much worse than before the pregnancy. I suffered from stomach pain with diarrhea, headache, back pain, serious fatigue, some irritability, sensitivity in food and smells, itching, flushing and so on. Always after my monthly period I had at least a week when I was more or less unable to do things at all. By the time I was supposed to go back to work it was no longer possible so I went into a long period of "sick leave". During this time I went to non-specialists that weren't very interested in my case. I got the feeling they thought I made it up.

After a while I finally went to see a doctor, this time I went to a dermatologist because of spots that I had all over my body which were itching terribly. I've had those spots since I was two years old but in my younger years I only had them on my feet. Now they were over my whole body, in some places very tightly together. The dermatologist easily saw that this was urticaria pigmentosa. When I told her about the other symptoms she told me that there was a systemic possibility when you had these spots on your skin but that she didn't think I had that since it was so uncommon. So I went away from there with just an antihistamine as a help for the itching. After some time I managed to persuade her to send me to a bone marrow biopsy which I finally did in December 2003 and I got the results in January 2004. The results were that my problem was mastocytosis.

The doctor who made the biopsy told me that I didn't need any medications for this and that I couldn't feel the way I did because of it. The dermatologist refused to send me to a specialist for mastocytosis since she had the same thoughts about how I felt as the biopsy doctor.

After doing my own research on the Internet (I naturally didn't feel any better just because they said I shouldn't feel the way I did), I found the names of a couple of doctors here that worked with patients with mastocytosis. One of the doctors in periods of the year worked very close by where I live. After finding these names and some information about mastocytosis I turned to a medical board which have as a task to make sure that people get the right treatment, they contacted my dermatologist and made sure she sent me to this specialist.

After waiting for about two months I finally got to see this doctor, Dr Hans Hagberg. He had a completely different view on how I could feel, since actually meeting some patients with this illness. After seeing him a couple of times he prescribed sodium cromoglicate to me. At first as a drinkable medication and then in addition as inhaled powder. It took about a month and I was feeling much better.

I was being able to have a social life and also going back to work - full time! I was very, very happy over this, especially since I never thought I'd be able to work again or even feel well again. I still had my symptoms but a lot less severe.

After talking to Dr Alan Edwards around the same time I went back to work we discussed me being in his study. I filled in the report cards daily for several months. During this period a pattern became very clear. During my monthly period I had about two weeks with a very high increase in symptoms and specially the fatigue. I got the advice to increase the inhaled sodium cromoglicate, which I did and my symptoms over all, including the fatigue, almost vanished.

With the help from Dr Alan Edwards, Dr Hans Hagberg and sodium cromoglicate I went from being unable to work and having a social life, to working full time and more, with traveling in work and a very rich social life. I still have minor symptoms off and on, and I still have fatigue around my monthly period but I can handle it now and keep it under control

## Consent

Written informed consent was obtained from the patient for publication of this case report and any accompanying images. A copy of the written consent is available for review by the Editor-in-Chief of this journal.

## Competing interests

AME was employed by the originators of sodium cromoglicate, Fisons Pharmaceuticals from 1974 to 1995. HH has no competing interest.

## Authors' contributions

AME conceived the study, participated in its design, and advised on the use of the sodium cromoglicate products and wrote the manuscript. HH saw the patient at a hospital clinic on a regular basis and controlled the treatment and investigations. Both authors and the patient read and approved the final manuscript.

## Supplementary Material

Additional file 1 **Shows copy of diary card used by subject to record severity of 20 symptoms each day**. Click here for file

## References

[B1] Castells MC Mastocytosis: classification, diagnosis, and clinical presentation Allergy Asthma Proc 2004 25 1 33 36 15055560

[B2] Cox JS Beach JE Blair AM Clarke AJ King J Lee TB Loveday DE Moss GE Orr TS Ritchie JT Sheard P Disodium cromoglycate (Intal) Adv Drug Res 1970 5 115 196 4099491

[B3] Aswania QA Corlett SA Chrystyn H Relative bioavailability of sodium cromoglycate to the lung following inhalation, using urinary excretion Br J Clin Pharmacol 1999 6 613 618 10.1046/j.1365-2125.1999.00937.x 10383538 PMC2014264

[B4] Walker SR Evans ME Richards AJ Paterson JW The fate of (14 C) disodium cromoglycate in man J Pharm Pharmacol 1972 24 7 525 531 4403844 10.1111/j.2042-7158.1972.tb09051.x

[B5] Dolovich J Punthakee ND MacMillan AB Osbaldeston GJ Systemic mastocytosis: control of lifelong diarrhea by ingested disodium cromoglycate Can Med Assoc J 1974 111 7 684 685 4213416 PMC1947871

[B6] Soter NA Austen KF Wasserman SI Oral disodium cromoglycate in the treatment of systemic mastocytosis N Engl J Med 1979 301 9 465 469 111124 10.1056/NEJM197908303010903

[B7] Czarnetzki BM Behrendt H Urticaria pigmentosa: clinical picture and response to oral disodium cromoglycate Br J Dermatol 1981 105 5 563 567 10.1111/j.1365-2133.1981.tb00800.x 6794592

[B8] Frieri M Alling DW Metcalfe DD Comparison of the therapeutic efficacy of cromolyn sodium with that of combined chlorpheniramine and cimetidine in systemic mastocytosis. Results of a double-blind clinical trial Am J Med 1985 78 1 9 14 10.1016/0002-9343(85)90454-1 3917606

[B9] Horan RF Sheffer AL Austen KF Cromolyn sodium in the management of systemic mastocytosis J Allergy Clin Immunol 1990 85 5 852 855 10.1016/0091-6749(90)90067-E 2110198

[B10] Basomba A Campos IG Villalmanzo Pelaez A The effect of sodium cromoglycate (SCG) in patients with food allergies Acta Allergol Suppl 1977 13 95 101 415503

[B11] Carini C Brostoff J Evidence for circulating IgE complexes in food allergy Ric Clin Lab 1987 17 4 309 322 3125594 10.1007/BF02886914

[B12] Villar SS Perez-Somarriba J Costa TS Winstanley S Escribano L Gonzalez B Interactive Case Report: A 38 year old woman with hypotensive shock at the onset of menstruation Br Med J 2009 338 654 655 10.1136/bmj.b868 19273521

[B13] Andre F Andre C Feknous M Colin L Cavagna S Digestive permeability to different-sized molecules and to sodium cromoglycate in food allergy Allergy Proc 1991 12 5 293 298 10.2500/108854191778879142 1959765

[B14] Auty RM Brown K Neale MG Snashall PD Respiratory tract deposition of sodium cromoglycate is highly dependent upon technique of inhalation using the Spinhaler Br J Dis Chest 1987 81 4 371 380 10.1016/0007-0971(87)90186-0 3130088

[B15] Gerth van Wijk R From statistical significance to clinical relevance Clin Experimental Allergy 2010 40 197 199 10.1111/j.1365-2222.2009.03422.x 20015275

