# Supplementary material for: Oral and inhaled sodium cromoglicate in the management of systemic mastocytosis: a case report
Source: J Med Case Rep. 2010 Jun 26;4:193. doi: 10.1186/1752-1947-4-193 (PMC2904792; doi:10.1186/1752-1947-4-193)
Supplement: Additional file 1 — Shows copy of diary card used by subject to record severity of 20 symptoms each day. [file 1752-1947-4-193-S1.DOC]

At the end of each day record the severity of the symptoms associated

**Diary Card** – Symptom profile and severity in systemic mastocytosis Date of starting this card …………………………

Patient Initials.…… ………… Date of Birth.,,,,,,,,,,,,,,,,,,,,,,,,,,,,

with your systemic mastocytosis experienced during the previous 24 hours.

Fill in one column each day. Use the following scale.

**0 = no symptoms 1 = mild 2 = moderate 3 = severe**

|  | **Date (mm/dd) →** |  |  |  |  |  |  |  |
| --- | --- | --- | --- | --- | --- | --- | --- | --- |
| Skin symptoms | Itching |  |  |  |  |  |  |  |
|  | Flushing |  |  |  |  |  |  |  |
|  | Whealing (hives) |  |  |  |  |  |  |  |
| Gastrointestinal symptoms | Abdominal pain |  |  |  |  |  |  |  |
|  | Diarrhoea |  |  |  |  |  |  |  |
|  | Nausea |  |  |  |  |  |  |  |
|  | Vomiting |  |  |  |  |  |  |  |
| Skeletal symptoms | Bone pain |  |  |  |  |  |  |  |
| Constitutional symptoms | Fatigue |  |  |  |  |  |  |  |
|  | Fever |  |  |  |  |  |  |  |
|  | Sweats |  |  |  |  |  |  |  |
| Cardiovascular symptoms | Palpitations |  |  |  |  |  |  |  |
|  | Fainting |  |  |  |  |  |  |  |
| Respiratory Symptoms | Cough |  |  |  |  |  |  |  |
|  | Wheeze |  |  |  |  |  |  |  |
|  | Chest Pain |  |  |  |  |  |  |  |
|  | Runny nose |  |  |  |  |  |  |  |
| Neurological symptoms | Headache |  |  |  |  |  |  |  |
|  | Decreased attention span |  |  |  |  |  |  |  |
|  | Difficulty in concentration |  |  |  |  |  |  |  |
|  | Irritability |  |  |  |  |  |  |  |
|  | Depression /lethargy |  |  |  |  |  |  |  |

**Diary Card Starting Date Patient Initials:**

**Current treatment.**

List all drugs being taken at present

Drug name Total daily dose

____________________________ ______________

____________________________ ______________

**Change in treatment**

Please make a note of any drugs stopped or new ones started whilst you are filling in this diary card

_________________________________________________________________

_________________________________________________________________
